# Person-Centered Health Care: An Approach That Integrates Acute and Long-Term Care

**DOI:** 10.1093/geroni/igaa057.2474

**Published:** 2020-12-16

**Authors:** Yuchi Young, Barbara Resnick

## Abstract

The world population is aging. The proportion of the population over 60 will nearly double from 12% in 2015 to 22% in 2050. Global life expectancy has more than doubled from 31 years in 1900 to 72.6 years in 2019. The need for long-term care (LTC) services is expanding with the same rapidity. A comprehensive response is needed to address the needs of older adults. Learning from health systems in other countries enables health systems to incorporate best long-term care practices to fit each country and its culture.

This symposium aims to compare long-term care policies and services in Taiwan, Singapore, and the USA where significant growth in aging populations is evidenced. In 2025, the aging population will be 20% in Taiwan, 20% in Singapore and 18 % in the USA. In the case of Taiwan, it has moved from aging society status to aged society, and to super-aged society in 27 years. Such accelerated rate of aging in Taiwan is unparalleled when compared to European countries and the United States. In response to this dramatic change, Taiwan has passed long-term care legislation that expands services to care for older adults, and developed person-centered health care that integrates acute and long-term care services. Some preliminary results related to access, care and patterns of utilization will be shared in the symposium. International Comparisons of Healthy Aging Interest Group Sponsored Symposium.

